# Stroke Event Factors among Adult Patients Admitted to Stroke Unit of Jimma University Medical Center: Prospective Observational Study

**DOI:** 10.1155/2019/4650104

**Published:** 2019-02-03

**Authors:** Ginenus Fekadu, Hunduma Wakassa, Firew Tekle

**Affiliations:** ^1^Department of Pharmacy, College of Health Science, Wollega University, Nekemte, Ethiopia; ^2^Jimma University Medical Center, Oromia, Jimma, Ethiopia; ^3^Department of Public Health, College of Health Science, Wollega University, Nekemte, Ethiopia

## Abstract

**Background:**

The fact that the majority of patients come late creates management difficulties as these first hours are important to avoid secondary insults to the brain and preserve the ischemic penumbra. Although thrombolytic treatments are currently not available in our hospital, significant delays during the prehospital or in-hospital phases of care create management difficulties and would make such advanced treatments impossible in the future in Ethiopia.

**Methods:**

Prospective observational study was carried at stroke unit of Jimma University Medical Center for 4 consecutive months from March 10 to July 10, 2017. Data was cleaned and entered to Epidata version 3.1 and then exported and analyzed using SPSS version 20.0.

**Results:**

A total of 116 eligible stroke patients were recruited during the study period with mean age of 55.1±14.0 years, ranging from 23 to 96 years. The majority of stroke patients were males (62.9%) and from rural areas (72.4%). The median time elapsed between the onset of stroke symptoms and arrival to the hospital was 27 hours. Almost half (47.4%) of the patients presented within 24 hours and 26 (22.4%) patients presented to hospital beyond 72 hours. Majority of patients (40.5%) showed severe neurological deficit on admission and the mean National Institute of health stroke scale (NIHSS) was 15.71 ± 7.52. The mean Glasgow coma scale (GCS) was moderate (12.12±3.35). On hospital arrival systolic blood pressure (SBP) was highly elevated (≥140 mm Hg) in 65.5% of the patients. The circadian pattern showed a significant peak in morning for hemorrhagic stroke (35.7%) and afternoon for ischemic stroke (38.3%).

**Conclusions:**

The delay of hospital arrival was a challenge similar to other high income countries for early management of the patients. Studies that attempt to determine some of the factors that impede timely presentation in patients with strokes are advisable to address those issues further.

## 1. Introduction

Cardiovascular disease is the primary global cause of death, accounting for more than 17.3 million deaths per year, a number that is expected to grow to more than 23.6 million by 2030. In 2013, cardiovascular deaths represented 31% of all global deaths, with 80% of those deaths taking place in low- and middle-income countries (LMICs) [1]. The data from the Global Burden of Diseases (GBD) showed that the leading cardiovascular diseases (CVD) cause of death and disability in 2010 in sub-Saharan Africa and other low- and middle-income countries (LMICs) was stroke [2–4].

First-time incidence of stroke occurs almost 17 million times a year worldwide, one every two seconds. Black people are twice as likely to have a stroke compared to white people [5]. It is an important disease worldwide, constituting a big burden on the public health purse as well as on patients and their relative [3, 4]. One in six people worldwide will have a stroke in their lifetime [4]. Patients with stroke under the age of 50 years account for 5-10% of all stroke worldwide [6]. It is the leading cause of acquired disability and the third leading cause of death in women worldwide [7].

Although age-standardized rates of stroke mortality have decreased worldwide in the past two decades, the absolute number of people who have a stroke every year, stroke survivors, and related deaths are high and sharply increasing in LMICs [8–10]. Its significance is likely to increase in the future due to ongoing demographic changes, including aging of the population and health transitions observed in developing countries [10]. Projections based on the current trends, incidence velocity, risk factor prevalence, population attributable risks, and relative risk for risk factors concluded that, by 2030, stroke will be the second leading cause of death globally and the first leading cause of death in LMICs [11].

According to WHO report of 2014 including all ages and sexes, in Ethiopia from the total cases of deaths, 30% were attributed to noncommunicable diseases (NCDs) and of these NCDs one-third of mortality was accounted by cardiovascular disease (CVD) [12]. Stroke is currently observed to be one of the commonest reasons of admission in many hospitals and becoming an increasingly serious public health issue in Ethiopia [13, 14]. It is an important health issue worldwide and the risk factor profile may vary with ethnicity, geographic region, age, gender, and stroke subtype [15]. Underdiagnosing of hypertension and other risk factors and delayed presentation at the hospital are the major challenges to address [13].

As patients usually present late and the standard of care is poor compared to hospitals in developed countries, the in-hospital mortality is expected to be higher [14]. Like other developing countries resources for stroke care and rehabilitation are deficient in Ethiopia [3]. Significant delays during the prehospital or in-hospital phases of care augmented by insufficient management of cases in the country are also making prognosis of stoke patients very poor. Contributing to this is the fact that thrombolytic therapy has been proven to be beneficial if administered within 4.5 hours after the onset of an ischemic stroke [3].

## 2. Methods and Participants

The study was conducted at stroke unit of Jimma University Medical Center (JUMC), a tertiary hospital found in Jimma city, southwest Ethiopia. Jimma is located 357km South West of the capital city of Ethiopia, Addis Ababa. JUMC has endured time as one of the oldest public hospitals in Ethiopia. Its history is traced back to the short-lived Italian occupation of the country, when it served as a medical center for soldiers. Shortly after, it was renamed as Ras Desta Damtew Hospital by the Imperial regime and then Jimma Hospital by the Derg. It has a total bed capacity around 555 with nearly 1600 hospital staffs.

The prospective observational study was carried out for 4 consecutive months from March 10 to July 10, 2017. All 116 stroke patients ≥ 18 years having either clinical diagnosis or confirmed by imaging as per WHO criteria for diagnosis of stroke [16] and admitted to stroke unit of JUMC within the study period were included. Patient or guardian not willing to give an informed consent, those who died before evaluation, if initial assessment or diagnosis of stroke was later changed to other cause (ruled out stroke) and patients with diagnosis of transient ischemic attack (TIA) and hematomas were excluded from the study [17].

### 2.1. Data Collection Tool and Procedure

A semistructured questionnaire containing the variables to be measured was designed, developed, and utilized based on the previous literature and using the WHO step wise approach to stroke surveillance along with different modifications and incorporations [18]. English, Afan Oromo, and Amharic version of the questioners was employed as the data collection tool. A formal document review indicating the aims and study process was provided to the facility to request the facility's permission. Two qualified nurses and one resident from the stroke unit of the hospital were trained to collect the relevant data from patient's cards and using interview. All relevant information about each patient such as sociodemographic characteristics and stroke events was recorded carefully.

### 2.2. Data Processing and Analysis

Checked data was cleaned and entered to Epidata version 3.1. Then, the data was exported and analyzed using SPSS version 20.0 for windows. Descriptive statistics such as percent, frequency, mean, and medians were used to summarize categorical variables of patients' characteristics and stroke event factors. The data analysis and correlation were done based on the selected variables for addressing all specific objectives adequately. The result was interpreted and presented using appropriate tables. Predictors with probability value less than 0.05 were considered statistically significant.

### 2.3. Operational Definition

*Stroke*: It is clinically defined as per WHO criteria, as rapidly developing clinical signs of focal or global disturbance of cerebral function, with symptoms lasting 24 hours or longer or leading to death, with no apparent cause other than vascular origin [7, 16, 19, 20].

*Ischemic Stroke*: It is evidence of a recent infarct in the clinically relevant area of the brain/confirmed cerebral infarction [15, 21]. 

*Hemorrhagic Stroke*: It occurs due to the weakening of blood vessel which would rupture and bleed into the surrounding brain tissues [22].

*Onset of Circadian Pattern of Stroke*

- **Morning**: 6 AM-11:59 AM;
- **Afternoon**: 12:00 PM -5:00 PM;
- ** Evening**: 5:01 PM- 8:00 PM;
- ** Night**: 8:01 PM- 5:59 AM.

*Episode /Type of Stroke Event [18]*

- **First ever: **people who have never had a stroke before either clinically or by neuroimaging. Previous TIA is not considered a stroke.
- **Recurrent:**
  - a history of a previous stroke event at some time in the past which meets the WHO definition or
  - a history of a new stroke event occurring more than 1 month after onset of a stroke event already registered.

**Glasgow coma scale:** it helps to measure level of consciousness [23].

- Good GCS (13-15): mild brain injury (alert).
- Moderate GCS (9-12): moderate brain injury (drowsy).
- Poor GCS (≤8): severe brain injury (unconscious).

**NIHSS**: it is tool used to evaluate stroke severity and the intervals were defined as [24].

- NIHSS 0–6 (mild).
- NIHSS 7–12 (moderate).
- NIHSS 13–20 (severe).
- NIHSS ≥ 21 (very severe).

## 3. Result

### 3.1. Sociodemographic Characteristics

The mean age of stroke patients was 55.1±14.0 years, ranging from 23 to 96 years. Stroke occurred at a mean age of 56.7 ±14.9 years in males and at 52.5 ±12.1 years in females. Stroke in the young, defined as age less than 45 years, accounted for 22.4% of the total patients, while 78.4% of the total patients were below 65 years of age. The majority of stroke patients were males (62.9%) and from rural areas (72.4%). Majority of the participants had informal education (able to read and write) 49 (42.2%) followed by no basic education (unable to write and read) 42 (36.2%). The mean body mass index (BMI) of the patient was 21.22±3.38 kg/m^2^ [17]. The median monthly income of the patients was $20 (ranged $8-40). Twenty patients (17.2%) were dependent and had no income. Hemorrhagic stroke was seen in 56 (48.3%) patients and ischemic stroke in 60 (51.7%) patients (Table 1).

### 3.2. Stroke Event Factors

The median time elapsed between the onset of stroke symptoms and arrival to the hospital was 27 hours (range 0 to 377 hours). The median time of arrival for IS and HS patients was 44 hours and 18.25 hours, respectively. Only nine patients presented within 3 hours of symptom onset and 14 patients (12.1%) presented within 4.5 hours (window period). Almost half (47.4%) of the patients presented within 24 hours and 26 (22.4%) patients presented to hospital beyond 72 hours. Compared to patients with ischemic stroke, patients with hemorrhagic stroke presented more often within 24 hours, 36.7% and 58.9%, respectively. There was no significant difference in terms of sex of the patient for hospital arrival. Additionally, the estimated median time of hospital arrival for urban residents was 18.25 hours and for rural residents 32.13 hours (P=0.953) (Table 2).

A major quota of patients (40.5%) showed severe neurological deficit (NIHSS 13–20) on admission (38.3% for ischemic stroke and 42.9% for hemorrhagic stroke). The mean NIHSS of the patients was 15.71 ± 7.52 and was significantly elevated in hemorrhagic stroke patients (17.54±7.54) compared to patients with ischemic strokes (14±7.15), (P=0.013).

The median time to CT scan imaging after hospital arrival was 4 days. Of the 61 patients with stroke confirmed by CT scan, 34.4 % were scanned within 3 days of hospital arrival, 50.8% between 3 days and 7 days, and 13.1% between 7 days and 14 days. Only 1 patient had CT scan 2 weeks after the hospital arrival [17].

The mean GCS of all stroke patients hospital arrival was moderate 12.12±3.35, for IS 13.07±2.56 and for HS 11.11±3.80 (P=0.002). About two-thirds (66.7%) of ischemic stroke patients had good GCS compared to 46.4% of HS patients. One-fourth of HS patients had severe brain injury as compared to 5% of IS patients (P=0.004) (Table 2).

The median systolic blood pressure and diastolic blood pressure of patients was 150 mmHg and 98.5 mmHg, respectively. The median SBP of IS and HS was 150 mmHg and 157 mmHg, respectively, and the median diastolic blood pressure of IS and HS was 90 mmHg and 100 mmHg, respectively. On hospital arrival majority of patients (65.5%) had systolic blood pressures (SBP) that was highly elevated (≥140 mm Hg), in particular 69.6% in hemorrhage stroke patients. The diastolic blood pressure was elevated (≥ 90 mm Hg) upon hospital arrival in 73 (62.9%) of the patients, in particular 71.4% of HS patients.

Majority of patients, 89.7%, presented with heart rate within normal range (60–100 bpm) and 10.3% had >100 bpm upon hospital arrival. The mean heart rate was 86±13 bpm for total patients and it was higher for IS as compared to HS patients (89±14 bpm versus 84±12 bpm) (P=0.037). A majority (71.6%) of patients presented with elevated respiratory rate (RR) (> 20 breath/min). The median RR was 24 breath/min for both ischemic and hemorrhagic stroke patients up on hospital arrival. Majority of the patients (83.6%) had normal temperature (36.0-37.1°C) up on hospital arrival.

The circadian pattern of stroke onset indicates that majority of stroke attack occur in the afternoon (32.8%) and in the morning (29.3%). This circadian pattern showed a significant peak in morning for hemorrhagic stroke (35.7%) and afternoon for ischemic stroke (38.3%). Out of total patients, stroke occurred in 35 (30.2%) while doing sedentary (nonsquatting) activities and in 27 (23.3%) while squatting. Majority of strokes occurred mostly in awake state (78.4%), particularly 83.9% of hemorrhagic stroke patients (P= 0.033).

Regarding stroke onset majority of the disease was associated with sudden onset in 88 (75.9%) patients. The fluctuating type of stroke was solely presented in 4 (6.7%) ischemic stroke patients. Of the 116 individuals with stroke, 107 (92.2%) suffered from first-event stroke episode, and the remaining 9 (7.8%) exhibited the recurrent/ previous stroke. The recurrent stroke was more significant in IS, 13.3% versus 1.8% (P=0.048). Two-thirds of patients with recurrent stroke were on treatment along with life style modification and the remaining were on life style modification only 3 (33.3%) (Table 2).

## 4. Discussion

The mean age of the patients (55.14±14.04 years) was in line with other studies in developing countries where the mean age of the patients diagnosed for stroke was between 50 and 60 years [23, 25–27], but lower by one decade in other studies which was between 60 and 70 years [28, 29]. This mean age was also in line with other studies in Ethiopia [24, 30]. The male predominance in stroke patients complies with other studies [4, 20, 24, 31, 32]. The possible explanation may be there is no vascular protection of endogenous estrogen in male.

The median time elapsed between the onset of stroke symptoms and the arrival to the hospital was 27 hours (IQR: 11.13-70.63 hours), which was delayed as compared to study in Gambia 8 hours [26], Brazil 12.9 hours [33], and Libya 12 hours [34] but earlier than in Senegal which was 2 days [29]. Cumulatively, only nine patients presented within 3 hours of symptom onset which correlates with other studies in Ethiopia, where less than 10 % of patients were admitted to the hospital within the first 3 hours [3, 13] and lower as compared to study in Brazil, a total of 22% of the patients were admitted within 3 hours from symptom onset [33]. In this study 14 patients (12.1%) presented within 4.5 hours which was lower compared to a study in Brazil 28.1% presenting within 4.5 hours [33]. Almost half of the patients (47.4%) patients presented within 24 hours which was higher than in Uganda 30% [35], other part of Ethiopia 31% [24], and Zambia 27.7% [36]. Forty patients (34.4%) patients presented to hospital beyond 48 hours. This delay complies with another study in Ethiopia in which 41.2% patients presented after 48 hours of symptom onset [3].

The fact that the majority of our patients come late creates management difficulties as these first hours are important to avoid secondary insults to the brain and preserve the ischemic penumbra. Considering the concept that “time is brain” these subsets of patients should have the acute treatment option with IV rTPA if available for them. The contraindication for this thrombolytic therapy was high because average delay in time of presentation to hospital was more than the intended window period in our study setting. Although thrombolytic treatment is currently not available in our country, significant delays during the prehospital or in-hospital phases of care create management difficulties and would make such advanced treatments difficult in the future even if rTPA is available in the local hospital. In addition to delayed presentation, brain imaging was delayed to a median of four days. Therefore, the prerequisite of receiving thrombolytic is not achieved and consequently rTPA was not given even to eligible patients.

Majority of patients (40.5%) showed severe neurological deficit on admission which was similar to study by Kuriakose et al. in India [22]. The mean NIHSS (15.71±7.52) on admission of the patients was higher than published studies by Nkoke et al. in Cameron [20] and Deresse et al. in Ethiopia [24]. The mean NIHSS was significantly higher in hemorrhagic stroke patients. This could be one possible reason for higher mortality of hemorrhagic stroke as compared to ischemic stroke patients. The mean Glasgow coma scale of the patients, 12.12±3.35, was lower compared to another study by Nkoke et al. [20].

During hospital arrival of the majority of patients (65.5%) systolic blood pressures (SBP) were highly elevated (≥140 mm Hg) that complies with another study by Gebremariam et al. [13]. The median SBP and DBP were higher for hemorrhagic stroke as compared to IS at hospital arrival which was statistical significant (P< 0.05) and similar to other study [23]. Most patients 104 (89.7%) presented with normal heart rate range (60–100 bpm) upon hospital arrival similar to finding by Gebremariam et al. [13], but median RR of 24 breath/min was higher than study by Gebremariam et al. [13]. Elevated respiratory rate may be related to comorbidities that may increase respiratory rate.

The circadian pattern of stroke onset indicates that majority of stroke attacks occur in the afternoon and in the morning which was consistent with study by Sarkar et al. in India [23]. This circadian pattern showed a significant peak in morning for hemorrhagic stroke and afternoon for ischemic stroke, which was contrary to a study in in India by Kuriakose et al. [22]. This could be correlated to biological factors such as blood pressure (with physiological nocturnal decrease and morning increase) and autonomic systemic activity (with activation of sympathetic nervous system after wake up movement with consequence on vascular tone, blood pressure, and heart rate). The endogenous factors also depend partially by the day-night cycle of the physical activity and assumption of the up-right posture (as exogenous factors) associated with awaking movement.

Majority of strokes occurred while patients were doing sedentary (nonsquatting) activities and while the patients were squatting which was unlike a study by Sarkar et al. in which majority of strokes occurred just after patients were awakening and while being involved in outdoor activities [23]. In this study majority of strokes occurred mostly in awake state (78.4%). The finding agreed with a study by Sarkar et al. in which 88% stroke events occurred in awake state [23]. This showed that there is diurnal variation and close relationship of stroke events with variation of activity.

Regarding stroke onset majority of the disease was associated with sudden onset in agreement with study by Ghandehari et al. in Iran [37]. Of the total patients, 7.8% exhibited recurrent/previous stroke, similar to a study by Alemayehu et al. in Ethiopia [3]. Of patients with recurrent stroke two-thirds of them were on treatment along with life style modification and the remaining were on life style modification only, in agreement with study by Alemayehu et al. [3].

### 4.1. Strength and Limitations of the Study

The major strength of this study was its prospective study design and the enrollment of consecutive patients. We have performed a detailed initial assessment including a NIHSS stroke scale in the hospital in a series of patients with stroke.

Regarding weaknesses of the study first, this was a hospital-based study rather than population based and hence may be subjected to referral bias. As our study ascertained events over a 4-month period, we acknowledge the possibility of a contribution of seasonal variation in stroke rates to our findings and were unable to analyze trends in stroke rates over time, as our study did not run for a complete 1-year period.

## 5. Conclusion 

The level of poor blood pressure control in hypertensive patients we observed in this study was alarming because in more than two-third of the patients, blood pressure was elevated (≥140/90 mm Hg) on hospital arrival. The delay of hospital arrival and imaging were challenges similar to other developing countries for early management and recovery of the patients. There was diurnal variation and close relationship of stroke events with variation of activity in this study. The circadian pattern onset of stroke showed a significant peak in morning for hemorrhagic stroke and afternoon for ischemic stroke. Majority of stroke onset was associated with sudden onset and first-ever stroke episode.

There should be aggressive propaganda from every social, media, and political level of the country in the purpose of increasing the awareness of risk factors and stroke events by making the people understand the devastating effect of the disease on human health and economy of the country. Additional studies that attempts to assess delays for treatment and to determine some of the factors that impede timely presentation in patients with strokes are advisable to address those issues further. Finally a prospective community based stroke incidence and prevalence studies are required to identify stroke event factors.

## Figures and Tables

**Table 1:** Sociodemographic characteristics of stroke among adult patients admitted to stroke unit of JUMC from March 10 to July 10, 2017.

**Sociodemographic factors**		**Frequency (n=116)**	**Percentage (**%**)**
*Age (years)*	< 45	26	22.4%
	45-65	65	56.0%
	>65	25	21.6%

*Sex*	*Male*	73	62.9%
	*Female*	43	37.1%

*Residence*	*Rural*	84	72.4%
	*Urban *	32	27.6%

*Marital status*	*Married*	104	89.7%
	*Widow*	11	9.5%
	*Divorced*	1	0.9%

*Education status*	*Unable to read and write*	42	36.2%
	*Able to read and write, informal education *	49	42.2%
	*Elementary school (1-8)*	17	14.7%
	*Secondary school (9-12)*	3	2.6%
	*College/university or above*	5	4.3%

*Occupational status (over the last 1 year)*	*Agriculture / farmer*	44	37.9%
	*Homemaker/ housewives *	41	35.3%
	*Merchant*	11	9.5%
	*Retired*	6	5.2%
	*Government employee*	5	4.3%
	*Other own business work*	5	4.3%
	*Skilled/unskilled manual labor/ daily worker*	4	3.4%

*Body mass index (BMI) (kg/m* ^*2*^)	*≤18.5 (underweight)*	24	20.7%
	*18.6–24.9 (normal)*	74	63.8%
	*25.0–29.9 (overweight)*	18	15.5%

* Approximated monthly income (Dollar)*	*None (dependent)*	20	17.2%
	*<20*	46	39.7%
	*20-40*	25	21.6%
	*>40*	25	21.6%

**Table 2:** Stroke event factors among adult stroke patients admitted to stroke unit of JUMC from March 10 and July 10, 2017.

**Stroke event factors**		**Total patients**	**Ischemic stroke**	**Hemorrhagic stroke**	**P value**
		**(N=116)**	**(n=60)**	**(n=56)**	**(OR)**
*Time interval from onset of stroke to hospital arrival (hours)*	*Median (IQR)*	27 (11.13-70.63)	44 (12.46-92.00)	18.25 (9.00-49.05)	0.059
	≤*4.5 hours*	14 (12.1%)	9 (15.0%)	5 (8.9%)	-
	*4.51-12 hours*	21 (18.1%)	6 (10.0%)	15 (26.8%)	0.042
	*12.01-24 hours*	20 (17.2%)	7 (11.7%)	13 (23.2%)	0.098
	*24.01-48 hours*	21 (18.1%)	12 (20.0%)	9 (16.1%)	0.693
	*>48 hours*	40 (34.5%)	26 (43.3%)	14 (25%)	0.962

*NIHSS score at hospital arrival*	*Mean *±* SD*	15.71±7.52	14±7.15	17.54±7.54	0.013
	*NIHSS 0–6 (mild)*	12 (10.3%)	11 (18.3%)	1 (1.8%)	-
	*NIHSS 7–12 (moderate)*	31 (26.7%)	17 (28.3%)	14 (25.0%)	0.046
	*NIHSS 13–20 (severe)*	47 (40.5%)	23 (38.3%)	24 (42.9%)	0.024
	*NIHSS ≥ 21 (very severe)*	26 (22.4%)	9 (15.0%)	17 (30.4%)	0.007

*GCS on hospital arrival*	*Mean*±* SD*	12.12±3.35	13.07±2.56	11.11±3.80	0.002
	*Poor GCS (≤8)*	17 (14.7%)	3 (5.0%)	14 (25.0%)	0.004
	*Moderate GCS/ (9-12) *	33 (28.4%)	17 (28.3%)	16 (28.6%)	0.389
	*Good GCS/ (13-15)*	66 (56.9%)	40 (66.7%)	26 (46.4%)	-

*Systolic BP at hospital arrival*	*Median (IQR)*	150 (130-180)	150 (124.25-170)	157 (130.75-189.25)	0.049
	*90–120 mm Hg*	21 (18.1%)	14 (23.3%)	7 (12.5%)	-
	*121–139 mm Hg *	19 (16.4%)	9 (15.0%)	10 (17.9%)	0.221
	*≥140 mm Hg *	76 (65.5%)	37 (61.7%)	39 (69.6%)	0.149

*Diastolic BP at hospital arrival*	*Median (IQR)*	98.50 (80-110)	90 (70.25-108.75)	100 (88-110)	0.047
	*≤59 mm Hg *	2 (1.7%)	2 (3.3%)	0 (0%))	0.999
	*60–80 mm Hg*	33 (28.4%)	21 (35.0%)	12 (21.4%)	-
	*81-89 mm Hg *	8 (6.9%)	4 (6.7%)	4 (7.1%)	0.999
	≥*90 mm Hg *	73 (62.9%)	33 (55.0%)	40 (71.4%)	0.999

*HR during hospital arrival*	*Mean*±* SD*	86.34±13.23	88.85±13.70	83.66±12.27	0.037
	*Normal 60–100 bpm *	104 (89.7%)	52 (86.7%)	52 (92.9%)	-
	*>100 bpm (tachycardia)*	12 (10.3%)	8 (13.3%)	4 (7.1%)	0.281

*RR during hospital arrival *	*Median (IQR)*	23.5 (20-24)	23.50 (20.25-24)	23.50 (20-26)	0.516
	*12–20 breath/min *	33 (28.4%)	15 (25.0%)	18 (32.1%)	-
	*> 20 breath/min *	83 (71.6%)	45 (75.0%)	38 (67.9%)	0.704

*Temp during hospital arrival*	*Median (IQR)*	36.6 (36.5-36.8)	36.6 (36.5-36.9)	36.6 (36.43-36.80)	0.666
	*<36*°*C*	3 (2.6%)	2 (3.3%)	1 (1.8%)	0.588
	*Normal (36.0–37.1*°*C) *	97 (83.6%)	49 (81.7%)	48 (85.7%)	-
	*>37.1*°*C (Hyperthermia)*	16 (13.8%)	9 (15.0%)	7 (12.5%)	0.739

*Onset of circadian pattern of stroke *	*In the morning*	34 (29.3%)	14 (23.3%)	20 (35.7%)	0.321
	*In the afternoon *	38 (32.8%)	23 (38.3%)	15 (26.8%)	0.103
	*At night*	28 (24.1%)	14 (23.3%)	14 (25.0%)	0.488
	*In the evening*	16 (13.8%)	9 (15.0%)	7 (12.5%)	-

*Activity of patient during stroke attack*	*At sleep*	25 (21.6%)	16 (26.7%)	9 (16.1%)	-
	*after awakening*	22 (19.0%)	11 (18.3%)	11 (19.6%)	0.897
	*at squatting*	27 (23.3%)	14 (23.3%)	13 (23.2%)	0.377
	*While doing sedentary activities*	35 (30.2%)	16 (26.7%)	19 (33.9%)	0.632
	*busy in household work *	2 (1.7%)	1 (1.7%)	1 (1.8%)	0.960
	*While involved in outdoor activities.*	5 (4.3%)	2 (3.3%)	3 (5.4%)	0.628

*Type of stroke onset*	*Fluctuating*	4 (3.4%)	4 (6.7%)	0 (0%)	0.999
	*Progressive*	24 (20.7%)	13 (21.7%)	11 (19.6%)	0.645
	*Sudden*	88 (75.9%)	43 (71.7%)	45 (80.4%)	-
*Episode of stroke event*	*Fist ever episode*	107 (92.2%)	52 (86.7%)	55 (98.2%)	-
	*Recurrent/ previous stroke*	9 (7.8%)	8 (13.3%)	1 (1.8%)	0.048

*∗*BP: blood pressure, GSC: Glasgow coma scale, HR: heart rate, NIHSS: National institute of health stroke scale, OR: Odds ratio, R: range, RR: respiratory rate, and SD: standard deviation.

## Data Availability

The data used to support the findings of this study are included within the article.

## Abbreviations

CVA: Cerebrovascular accident
CVDs: Cardiovascular diseases
GBD: Global Burden of Diseases
GCS: Glasgow coma scale
HS: Hemorrhagic stroke
HTN: Hypertension
ICH: Intracerebral hemorrhage
IHD: Ischemic heart disease
IS: Ischemic stroke
JUMC: Jimma University Medical Center
LMICs: Low- and middle-income countries
NCDs: Noncommunicable diseases
NIHSS: National Institute of Health Stroke Scale
rTPA: Recombinant tissue plasminogen activator
SSA: Sub-Saharan Africa
SU: Stroke unit
WHO: World Health Organization.

## References

[1] Mozaffarian D., Benjamin E. J., Go A. S. (2016). Heart disease and stroke statistics-2016 update: a report from the american heart association. *Circulation*.

[2] Moran A., Forouzanfar M., Sampson U., Chugh S., Feigin V., Mensah G. (2013). The epidemiology of cardiovascular diseases in sub-Saharan Africa: the Global Burden of Diseases, Injuries and Risk Factors 2010 Study. *Progress in Cardiovascular Diseases*.

[3] Alemayehu C. M., Birhanesilasie S. K. (2013). Assessment of stoke patients: occurrence of unusually high number of haemorrhagic stroke casesin tikur anbessa specialized hospital, Addis Ababa, Ethiopia. *Clinical Medicine Research*.

[4] Patne S., Chintale K. (2016). Study of clinical profile of stroke patients in rural tertiary health care centre. *International Journal of Advances in Medicine*.

[5] United kingdom. State of the Nation Stroke statistics 2016, https://www.stroke.org.uk

[6] Tan K. S., Navarro J. C., Wong K. S. (2014). Clinical profile, risk factors and aetiology of young ischaemic stroke patients in Asia: A prospective, multicentre, observational, hospital-based study in eight cities. *Neurology Asia*.

[7] Manorenj S., Inturi S., Jyotsna B., Savya V., Areli D., Reddy B. (2016). Prevalence, pattern, risk factors and outcome of stroke in women: a clinical study of 100 cases from a tertiary care center in South India. *International Journal of Research in Medical Sciences*.

[8] Krishnamurthi R. V., Feigin V. L., Forouzanfar M. H. (2013). Global and regional burden of first-ever ischaemic and haemorrhagic stroke during 1990–2010: findings from the Global Burden of Disease Study 2010. *The Lancet Global Health*.

[9] Feigin V. L., Forouzanfar M. H., Krishnamurthi R. (2014). Global and regional burden of stroke during 1990–2010: findings from the Global Burden of Disease Study 2010. *The Lancet*.

[10] Feigin V. L., Krishnamurthi R. V., Parmar P. (2015). Update on the global burden of ischemic and hemorrhagic stroke in 1990-2013: The GBD 2013 study. *Neuroepidemiology*.

[11] Owolabi M. O., Akarolo-Anthony S., Akinyemi R. (2015). The burden of stroke in Africa: A glance at the present and a glimpse into the future. *Cardiovascular Journal of Africa*.

[12] World Health Organization Non-communicable diseases (NCD) country profile of 2014 Geneva. http://apps.who.int/iris/bitstream/10665/128038/1/9789241507509_eng.pdf?ua=1.

[13] Gebremariam S. A., Yang H. S. (2016). Types, risk profiles, and outcomes of stroke patients in a tertiary teaching hospital in northern Ethiopia. *eNeurologicalSci*.

[14] Greffie E. S., Mitiku T., Getahun S. (2015). Risk Factors, Clinical Pattern and Outcome of Stroke in a Referral Hospital, Northwest Ethiopia. *Clinical Medicine Research*.

[15] Wu C. Y., Wu H. M., Lee J. D., Weng H. H. (2010). Stroke risk factors and subtypes in different age groups: A hospital-based study. *Neurology India*.

[16] Ekeh B., Ogunniyi A., Isamade E., Ekrikpo U. (2015). Stroke mortality and its predictors in a Nigerian teaching hospital. *African Health Sciences*.

[17] Fekadu G., Chelkeba L., Melaku T., Tegene E. (2018). Pathological sub types and diagnostic protocols of stroke among adult patients admitted to jimma university medical center, South West Ethiopia. *Journal of Neurology & Neurophysiology*.

[18] World Health Organization The WHO step wise approach to stroke surveillance Geneva 2006. http://www.who.int/chp/steps/Stroke/en/.

[19] Jowi J. O., Mativo P. M. (2008). Pathological sub-types, risk factors and outcome of stroke at the Nairobi hospital, Kenya. *East African Medical Journal*.

[20] Nkoke C., Lekoubou A., Balti E., Kengne A. P. (2015). Stroke mortality and its determinants in a resource-limited setting: A prospective cohort study in Yaounde, Cameroon. *Journal of the Neurological Sciences*.

[21] Dash D., Bhashin A., Pandit A. k. (2014). Risk Factors and Etiologies of Ischemic Strokes in Young Patients: A Tertiary Hospital Study in North India. *Journal of Stroke*.

[22] Kuriakose C., Shifafiya M. N., Tharakan N. S., Sattanathan K., Kumar R. S. (2016). A prospective study of clinical profile of stroke in a tertiary care hospital. *Asian Journal of Pharmaceutical and Clinical Research*.

[23] Sarkar D., Halder S., Saha B., Biswas P. (2016). A study of stroke patients with respect to their clinical and demographic profile and outcome. *International Journal of Research in Medical Sciences*.

[24] Deresse B., Shaweno D. (2015). Epidemiology and in-hospital outcome of stroke in South Ethiopia. *Journal of the Neurological Sciences*.

[25] Watila M. M., Nyandaiti Y. W., Ibrahim A. (2012). Risk factor profile among black stroke patients in Northeastern Nigeria. *Journal of Neuroscience and Behavioural Health*.

[26] Walker R. W., Rolfe M., Kelly P. J., George M. O., James O. F. (2003). Mortality and recovery after stroke in the Gambia. *Stroke*.

[27] Masood C. T., Hussain M., Anis ur R., Abbasi S. (2013). Clinical presentation, risk factors and outcome of stroke at a district level teaching hospital. *Journal of Ayub Medical College Abbottabad*.

[28] Tirschwell D. L., Ton T. G., Ly K. A. (2012). A prospective cohort study of stroke characteristics, care, and mortality in a hospital stroke registry in Vietnam. *BMC Neurology*.

[29] Sagui E., M'Baye P. S., Dubecq C. (2005). Ischemic and hemorrhagic strokes in Dakar, Senegal: A hospital-based study. *Stroke*.

[30] Bekele A., Kebede O. (2002). Stroke admission to Tikur Anbessa Teaching Hospital: with emphasis on stroke in the young. *Ethiopian Journal of Health Development*.

[31] Vaidya C., Majmudar D. (2014). A retrospective study of clinical profile of stroke patients from GMERS Medical College and Hospital, Gandhinagar, Gujarat. *International Journal of Clinical Trials*.

[32] Owolabi M. O., Agunloye A. M. (2013). Which risk factors are more associated with ischemic rather than hemorrhagic stroke in black Africans?. *Clinical Neurology and Neurosurgery*.

[33] de Carvalho J. J. F., Alves M. B., Viana G. Á. A. (2011). Stroke epidemiology, patterns of management, and outcomes in Fortaleza, Brazil: a hospital-based multicenter prospective study. *Stroke*.

[34] Bennour A., Ehmoda F., Bo-Shaala S. (2014). Clinical characteristics, management and outcome of patients admitted with acute stroke in Benghazi, Libya. *Ibnosina Journal of Medicine and Biomedical Sciences*.

[35] Nakibuuka J., Sajatovic M., Nankabirwa J. (2015). Early mortality and functional outcome after acute stroke in Uganda: prospective study with 30 day follow-up. *SpringerPlus*.

[36] Atadzhanov M., Mukomena P., Lakhi S., Ross O. A., Meschia J. F. (2012). Stroke characteristics and outcomes of adult patients admitted to the university teaching hospital, Lusaka, Zambia. *The Open General and Internal Medicine Journal*.

[37] Ghandehari K., Izadi-Mood Z. (2007). Khorasan stroke registry: analysis of 1392 stroke patients. *Archives of Iranian medicine*.

